# Chronotype and Subjective Memory Complaints: The Sequential Mediating Roles of Sleep Quality and Psychological Distress

**DOI:** 10.3390/bs16030457

**Published:** 2026-03-19

**Authors:** Pedro F. S. Rodrigues, Marco Lopes, Inês B. Oliveira, Sara M. Fernandes, Ana Bártolo, Ana Paula Caetano, Ramón López-Higes, Susana Rubio-Valdehita, Pedro B. Albuquerque

**Affiliations:** 1 RISE-Health (RISE-Health@UPT), Portucalense University, 4200-072 Porto, Portugal; 2 Department of Psychology and Education, Portucalense University, 4200-072 Porto, Portugal; 3 Intrepid Lab, Lusófona University, 4000-098 Porto, Portugal; 4 CLISSIS, Lusiada Research Center on Social Work and Social Intervention, 1349-001 Lisboa, Portugal; 5 Departamento de Psicología Experimental, Complutense University of Madrid, 28223 Madrid, Spain; 6 Departamento de Psicología Social, del Trabajo y Diferencial, Complutense University of Madrid, 28223 Madrid, Spain; 7 CIPsi (Psychology Research Centre, School of Psychology), University of Minho, 4710-057 Braga, Portugal

**Keywords:** chronotype, subjective memory complaints, sleep quality, psychological distress

## Abstract

Individual differences in circadian preference have been shown to influence cognitive functioning, yet their relationship with subjective memory complaints remains unclear. The present study examined the association between chronotype and everyday memory complaints in a sample of Portuguese adults, exploring the sequential mediating roles of sleep quality and psychological distress. A total of 382 participants completed self-report measures of chronotype, sleep quality, psychological distress (anxiety, depression, and stress), and subjective memory complaints. In a cross-sectional self-report design, a path analysis approach was used to test a theoretically driven serial mediation model. Results indicated that greater morningness predicted better perceived sleep quality, which in turn was associated with lower levels of psychological distress. No significant direct effects of chronotype or sleep quality on subjective memory complaints were observed; however, a significant indirect effect was identified through the sequential pathway linking chronotype, sleep quality, and psychological distress. These findings suggest that circadian preferences are associated with self-perceived memory functioning primarily through sleep-related and emotional mechanisms; however, the sequential mediation identified reflects associational rather than causal relationships. The model highlights the central role of sleep quality and emotional state in shaping subjective memory complaints and supports integrative approaches that consider both circadian and emotional factors.

## 1. Introduction

Circadian rhythms are endogenous biological oscillations that occur in approximately 24 h cycles, enabling the synchronization of internal physiological processes with environmental cues such as the light–dark cycle (Adan et al., 2012; Bauducco et al., 2020; Halberg, 1963). These rhythms play a central role in regulating the sleep–wake cycle, hormonal secretion, body temperature, and metabolic processes, thereby contributing to the maintenance of homeostatic balance (Adan et al., 2012; Cox et al., 2024; Gao et al., 2019; Lamprou et al., 2024; Minz & Pati, 2021; Roenneberg et al., 2019; Schmidt et al., 2007).

Beyond their physiological functions, circadian rhythms influence daily fluctuations in physical performance, emotional regulation, and cognitive functioning, including attention and memory processes (Chauhan et al., 2024; Heimola et al., 2021; Montaruli et al., 2021; Oliveira et al., 2024; Schmidt et al., 2007). Importantly, these fluctuations are not constant across individuals but are modulated by interindividual differences in chronotype^1^, commonly conceptualized along the morningness–eveningness continuum (Mecacci et al., 2004; Sabaoui et al., 2023).

Chronotype refers to an individual’s predisposition toward a preferred timing of sleep and wakefulness and represents the phenotypic expression of circadian phase variability (Adan et al., 2012; Bauducco et al., 2020; Horne & Östberg, 1977). Chronotype emerges from the interaction between genetic factors—particularly clock-related genes—and environmental influences such as light exposure and social schedules, with the suprachiasmatic nucleus of the hypothalamus acting as the central circadian pacemaker (Adan et al., 2012; Roenneberg et al., 2019). These individual differences in circadian timing have been shown to influence cognitive performance across the day, particularly in domains such as executive functions and working memory (Heimola et al., 2021; Schmidt et al., 2007).

Morning-type individuals tend to fall asleep and wake earlier, report greater alertness and cognitive efficiency during the early hours of the day, and show an earlier peak in melatonin secretion, which facilitates sleep initiation and daytime functioning (Adan et al., 2012; Montaruli et al., 2021; Taylor & Hasler, 2018). In contrast, evening-type individuals display a delayed circadian phase, characterized by later sleep and wake times and peak alertness in the late afternoon or evening. This misalignment between biological rhythms and socially imposed schedules often results in sleep restriction, poor sleep quality, and increased vulnerability to mental health difficulties (Adan et al., 2012; Montaruli et al., 2021; Zou et al., 2022; Zhao et al., 2025). Intermediate-type individuals, who constitute most of the population, exhibit greater flexibility in sleep–wake timing and less pronounced circadian preferences (Heimola et al., 2021; Schmidt et al., 2007).

Differences in circadian phase among chronotypes have important implications for sleep regulation and emotional functioning (Adan et al., 2012; Montaruli et al., 2021; Morris & Kountouriotis, 2024). In this context, sleep quality emerges as a key mechanism linking chronotype to both cognitive and emotional outcomes (e.g., Montaruli et al., 2021). Beyond simple behavioral preferences, chronotype reflects underlying neurobiological processes involving the suprachiasmatic nucleus, hormonal rhythms, and sleep architecture. Circadian misalignment, particularly among evening-type individuals exposed to socially imposed schedules, has been associated with alterations in melatonin onset, dysregulated cortisol rhythms, and increased activation of the hypothalamic–pituitary–adrenal axis. These processes may contribute to fragmented sleep and reduced slow-wave sleep, a stage critically involved in memory consolidation and emotional regulation. Consequently, circadian misalignment may indirectly influence cognitive functioning by disrupting restorative sleep mechanisms and increasing vulnerability to emotional distress (e.g., Dollish et al., 2024).

Sleep quality is a critical determinant of cognitive performance, emotional regulation, and overall well-being. Disrupted sleep has been associated with attentional deficits, impaired memory functioning, and increased daytime sleepiness, ultimately compromising daily functioning and quality of life (Pavlova & Latreille, 2019). Empirical evidence consistently shows that poor sleep quality is associated with increased subjective memory complaints and heightened psychological distress, including symptoms of anxiety, depression, and stress (Basagni & Navarrete, 2025; Gonzales et al., 2023; Scott et al., 2021; Simor et al., 2015). Chronotype is closely linked to sleep quality, with evening-type individuals more likely to experience fragmented sleep and insufficient sleep duration, partly due to increased exposure to artificial light at night and social jetlag (Akram et al., 2019; Roenneberg et al., 2019). These sleep-related disruptions may, in turn, exacerbate emotional dysregulation. Anxiety and depressive symptoms have been repeatedly associated with eveningness, reflecting difficulties in adapting to conventional social and occupational demands and increased vulnerability to emotional distress (Antypa et al., 2016; Druiven et al., 2019; Morris & Kountouriotis, 2024).

While not all evening-type individuals develop clinical psychopathology, subclinical levels of distress are common and can negatively affect cognitive and emotional functioning (American Psychiatric Association, 2022; Scott et al., 2021). Importantly, sleep quality appears to play a central role in this association, acting as a pathway through which chronotype-related misalignment contributes to emotional distress and subjective memory complaints.

Investigating subjective memory complaints within a circadian framework is particularly relevant because these complaints frequently emerge in non-clinical populations and may reflect modifiable affective and sleep-related mechanisms rather than irreversible cognitive decline (e.g., Csábi et al., 2025). Nevertheless, evidence suggests that these complaints are frequently more closely related to emotional factors, including symptoms of depression and anxiety, as well as poor sleep quality, than to objective mnemonic dysfunction. In many cases, individuals reporting memory complaints show preserved performance on standardized neuropsychological memory measures, indicating that subjective perceptions of memory decline may reflect affective distress and sleep-related difficulties rather than measurable impairments in memory functioning (e.g., Dzierzewski, 2022). From a psychological perspective, sleep problems and emotional dysregulation may shape individuals’ subjective perceptions of cognitive functioning through metacognitive and affective pathways. Elevated anxiety and depressive symptoms are known to bias self-evaluation processes, increase rumination, and heighten attentional focus on perceived cognitive failures. In this context, subjective memory complaints may reflect an interaction between sleep-related cognitive fatigue and negative emotional appraisal rather than objective mnemonic impairment. This integrative framework suggests that chronotype may exert distal effects on subjective memory functioning through sleep quality and emotional distress as intermediate mechanisms (e.g., Csábi et al., 2025; Fusz et al., 2025).

Although previous studies have independently examined associations between chronotype and mental health (e.g., Montaruli et al., 2021), sleep quality and emotional distress (e.g., Lamprou et al., 2024), and sleep problems and subjective memory complaints (e.g., Dzierzewski, 2022), these constructs have rarely been integrated within a unified explanatory framework. Specifically, subjective memory complaints have received limited attention in circadian research, especially among young adult populations in which such complaints are often driven by emotional and contextual factors rather than neurodegenerative processes. By testing a serial mediation model linking chronotype, sleep quality, psychological distress, and subjective memory complaints, the present study addresses an important theoretical gap and contributes to a more comprehensive understanding of how circadian preferences translate into everyday cognitive experiences.

Building on this framework, the present study examines the associations between chronotype, sleep quality, psychological distress (anxiety, depression, and stress), and subjective memory complaints in a sample of Portuguese adults. Furthermore, it investigates whether sleep quality and psychological distress act as sequential mediators in the relationship between chronotype and subjective memory complaints. Based on this body of evidence, the proposed sequential model reflects a theoretically informed and temporally coherent ordering in which circadian predispositions influence sleep processes, which in turn shape emotional functioning and subjective cognitive appraisal. The present study formulated the following hypotheses. First, chronotype was expected to be associated with sleep quality, with greater eveningness predicting poorer perceived sleep quality (H1). Second, poorer sleep quality was expected to be associated with higher levels of psychological distress (H2). Third, psychological distress was hypothesized to be positively associated with subjective memory complaints (H3). Finally, it was hypothesized that chronotype would exert an indirect effect on subjective memory complaints through a sequential mediation pathway involving sleep quality and psychological distress (H4). Given inconsistent findings in prior literature, no specific hypothesis was formulated regarding a direct association between chronotype and subjective memory complaints.

## 2. Materials and Methods

### 2.1. Participants

Inclusion criteria were: age 18 years or older, fluency in European Portuguese, the absence of a current diagnosed mental disorder, and provision of informed consent.

A total of 625 responses were initially collected via an online protocol of questionnaires administered to native Portuguese speakers. Of these, 400 participants completed the full assessment protocol. Of them, eighteen cases were excluded due to inconsistent, implausible, or uninterpretable responses (e.g., reporting “730 hours” of effective sleep in the previous month). No cases were excluded due to age below 18, lack of fluency, absence of consent, or current mental disorder diagnosis.

The final sample consisted of 382 Portuguese participants aged 18 to 61 years. See Table 1 for sociodemographic characteristics.

### 2.2. Instruments

#### 2.2.1. Sociodemographic Questionnaire

A sociodemographic questionnaire specifically developed for the present study was used to characterize the sample in terms of personal, academic, and health-related variables. Collected information included sex, age, educational achievement, and fluency in European Portuguese.

#### 2.2.2. Morningness–Eveningness Questionnaire (MEQ)

The European Portuguese version of the MEQ (da Silva et al., 2002), adapted from the original instrument by Horne and Östberg (1976), consists of 16 items (e.g., “Considering only your personal well-being and having complete freedom to plan your evening, at what time would you go to bed?”). The questionnaire is designed to assess individual chronotype, allowing participants to be classified as morning-type, evening-type, or intermediate-type^2^. Items employ different response formats, including Likert-type scales ranging from 0 to 5, with higher scores indicating a tendency toward morningness.

In the present study, the internal consistency of the MEQ was assessed using Cronbach’s alpha (α), yielding a value of α = 0.83, which indicates good reliability in the current sample. This value exceeds that reported in the Portuguese validation study (α = 0.75; da Silva et al., 2002).

#### 2.2.3. Everyday Memory Questionnaire (EMQ-13)

The European Portuguese version of the EMQ-13 (Rodrigues et al., 2025), adapted from the original version by Royle and Lincoln (2008), comprises 13 items (e.g., “Completely forgetting to do something you said you would do or had planned to do”). The instrument assesses subjective complaints of everyday episodic memory functioning. Participants report how frequently each situation occurred during the previous month using a 5-point Likert-type scale ranging from 0 (once or less in the past month) to 4 (once or more per day). Higher scores reflect a greater frequency of subjective memory complaints.

In the present sample, the EMQ-13 demonstrated high internal consistency (α = 0.89), closely approximating the reliability reported in the Portuguese validation study (α = 0.92; Rodrigues et al., 2025).

#### 2.2.4. Depression, Anxiety, and Stress Scale–21 (DASS-21)

The European Portuguese version of the DASS-21 (Pais-Ribeiro et al., 2004), adapted from the original scale by Lovibond and Lovibond (1995), consists of 21 items (e.g., “I found it difficult to relax”), evenly distributed across three subscales: depression (items 3, 5, 10, 13, 16, 17, and 21), anxiety (items 2, 4, 7, 9, 15, 19, and 20), and stress (items 1, 6, 8, 11, 12, 14, and 18). The scale assesses the frequency of negative emotional symptoms experienced during the previous week using a 4-point Likert-type scale ranging from 0 (did not apply to me at all) to 3 (applied to me most of the time). Higher scores indicate greater symptom severity.

In this study, internal consistency was high for all subscales, with Cronbach’s alpha values of 0.90 for depression, 0.87 for anxiety, and 0.89 for stress. These values are comparable to those reported in the Portuguese validation study (Pais-Ribeiro et al., 2004).

#### 2.2.5. Pittsburgh Sleep Quality Index (PSQI)

The European Portuguese version of the PSQI (Gomes et al., 2018), adapted from the original instrument by Buysse et al. (1989), consists of 10 items (e.g., “During the past month, what time have you usually gone to bed at night?”) and is designed to assess sleep quality. The questionnaire includes objective items (e.g., bedtime, sleep latency, wake-up time, and total sleep duration), as well as subjective items assessing the frequency and severity of sleep disturbances. Responses are provided using different formats, including Likert-type scales ranging from 0 to 3. Higher total scores indicate poorer sleep quality.

In the present study, the PSQI showed adequate internal consistency (α = 0.75), identical to that reported in the Portuguese validation study (Gomes et al., 2018).

### 2.3. Procedures

The study was conducted in accordance with the ethical principles for scientific research outlined in the Declaration of Helsinki (World Medical Association, 2013), the Oviedo Convention (Council of Europe, 2001), and the Code of Ethics of the Portuguese Psychologists Association (Portuguese Psychologists Association, 2024). The study also complied with the General Data Protection Regulation (GDPR; European Parliament & Council of the European Union, 2016), ensuring the protection of participant privacy, data security, and confidentiality.

Data were collected online using the LimeSurvey platform (LimeSurvey GmbH, 2022). Participants were recruited through institutional email lists, social media platforms, and direct academic contacts, using a standardized recruitment message that described the study objectives and provided access to the questionnaire.

Before completing the questionnaires, participants were presented with a digital informed consent form detailing the study objectives, the voluntary nature of participation, the right to withdraw at any time without penalty, and assurances of anonymity and confidentiality. Access to the questionnaires was granted only after informed consent had been provided.

The assessment protocol consisted of the sociodemographic questionnaire followed by the validated instruments for the Portuguese population, presented in the same order to all participants. The average completion time was approximately 20 min.

Statistical analyses were conducted using IBM SPSS Statistics (Version 29; IBM Corp., 2023). Specifically, path analysis was conducted using maximum likelihood estimation in IBM SPSS Amos (Version 28). Psychological distress was modeled as a latent variable composed of anxiety, depression, and stress indicators from the DASS-21. Indirect effects were tested using bias-corrected bootstrapping procedures (5000 resamples), and 95% confidence intervals were computed. Model fit was evaluated using multiple indices, including the chi-square statistic (χ^2^), χ^2^/df ratio, Comparative Fit Index (CFI), Root Mean Square Error of Approximation (RMSEA) with 90% confidence interval, and Standardized Root Mean Square Residual (SRMR). Conventional cut-off criteria were considered (CFI ≥ 0.95; RMSEA ≤ 0.08; SRMR ≤ 0.08).

## 3. Results

### 3.1. Descriptive Statistics

The main sociodemographic and chronotype characteristics of the sample (*N* = 382) are summarized in Table 1. Means, standard deviations, and observed ranges for the main study variables are presented in Table 2. Higher scores on the chronotype measure indicate a tendency toward morningness. Higher scores on the sleep quality index reflect poorer perceived sleep quality. Higher scores on DASS-21 indicate greater levels of psychological symptomatology (anxiety, depression, and stress). Higher scores on the subjective memory complaints measure reflect more frequent everyday memory difficulties.

Participants ranged in age from 18 to 61 years (*M* = 23.14, *SD* = 7.23). Although the absence of a current diagnosed mental disorder was an inclusion criterion, 93 participants (24.30%) reported a past mental health diagnosis for which treatment had been completed. To examine whether prior mental health history influenced the results, supplementary sensitivity analyses were conducted excluding these participants. The pattern and significance of the findings remained unchanged, indicating that the results were not driven by this subgroup.

### 3.2. Preliminary Analyses

The normality of the variables was assessed through graphical inspection (histograms and Q–Q plots), skewness and kurtosis coefficients (see Table 2), and Kolmogorov–Smirnov tests with Lilliefors correction. The test results indicated statistically significant deviations from normality for all variables (*p* < 0.001), except for chronotype in the Kolmogorov–Smirnov test (*p* = 0.060). This pattern is expected in large samples, as formal normality tests become highly sensitive to minimal deviations. However, the skewness and kurtosis values and the graphical inspection did not suggest substantial departures from normality, with all absolute skewness and kurtosis values below 3 (Byrne, 2016). Thus, considering the sample size (*N* = 382) and the robustness of parametric tests to moderate violations of normality, parametric analyses were retained, consistent with the Central Limit Theorem. To further enhance the robustness of the estimates, the mediation analysis employed bootstrapped confidence intervals (5000 samples), an approach that does not assume normality of the error distribution and provides more stable estimates of indirect effects.

### 3.3. Correlational Analyses

Pearson correlation coefficients among the main variables are presented in Table 3. Chronotype was negatively and significantly correlated with sleep quality, depressive symptoms, and stress, indicating that a more evening-oriented chronotype was associated with poorer sleep quality and higher levels of depressive and stress-related symptomatology. No significant associations were observed between chronotype and anxiety or subjective memory complaints. Sleep quality was positively and significantly associated with all distress-related variables (anxiety, depression, and stress), as well as with subjective memory complaints, suggesting that poorer sleep quality co-occurred with greater psychological distress and more frequent perceived memory difficulties. All psychological variables showed positive and significant intercorrelations, highlighting the strong interrelationship between emotional distress and subjective cognitive complaints. Based on conventional effect size benchmarks (Cohen, 1988), correlation coefficients around 0.10 were interpreted as small, around 0.30 as moderate, and 0.50 or above as large, suggesting that the associations observed in the present study were primarily small to moderate in magnitude.

### 3.4. Path Analysis: Serial Mediation Model

The pattern of bivariate associations was consistent with the hypothesized sequential structure, supporting the inclusion of indirect pathways in the path model among chronotype, sleep quality, distress (modeled as a latent construct comprising anxiety, depression, and stress), and subjective memory complaints. A path analysis was conducted to test this model and evaluate a serial mediation hypothesis.

Model fit indices indicated an overall good fit to the data. The chi-square test was significant, χ^2^(6) = 22.30, *p* = 0.001, a result likely influenced by the sensitivity of this statistic to sample size. The χ^2^/df ratio was 3.72, which falls within acceptable limits. The Comparative Fit Index (CFI) was 0.98, exceeding recommended thresholds for good model fit. The Root Mean Square Error of Approximation (RMSEA) was 0.084, with a 90% confidence interval of [0.049, 0.123] and a *p*-value of 0.055 for the test of close fit. Although slightly above the conventional 0.08 threshold, the confidence interval included values indicative of acceptable fit, and all other fit indices supported a well-fitting model. The Standardized Root Mean Square Residual (SRMR) was 0.030, indicating a lower level of residual error.

Chronotype exerted a significant direct effect on sleep quality, such that greater morningness was associated with better perceived sleep quality. No significant direct effect of chronotype on distress was observed. Sleep quality, however, emerged as a significant predictor of distress, with poorer sleep quality associated with higher levels of psychological distress.

No significant direct effects of chronotype or sleep quality on subjective memory complaints were identified. Nonetheless, statistically significant indirect effects emerged. Specifically, chronotype influenced subjective memory complaints indirectly through a sequential pathway involving sleep quality and psychological distress. This pattern supports a serial mediation model, whereby chronotype affects sleep quality, which in turn influences distress, ultimately contributing to greater subjective memory complaints.

**Figure 1:**
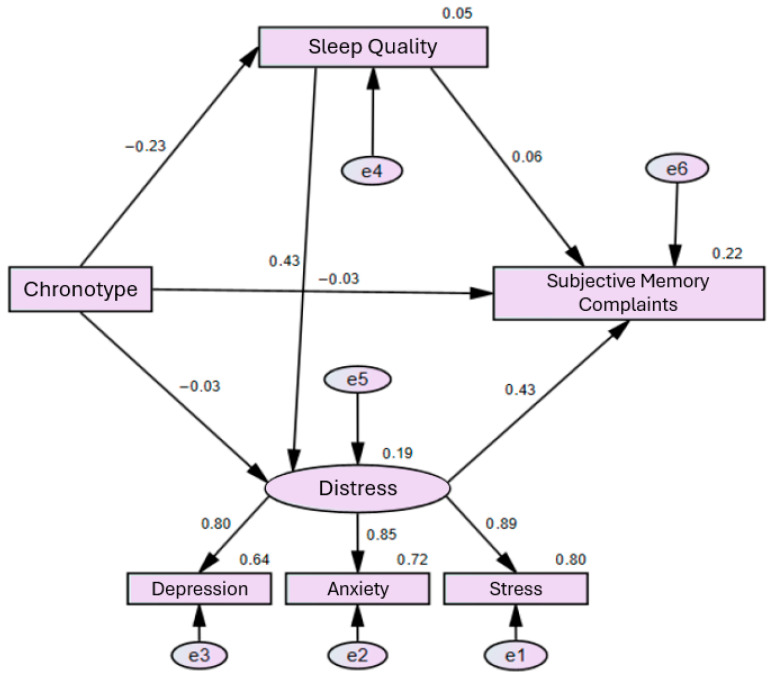
Serial Mediation Model.

Collectively, these findings support an explanatory framework in which sleep quality and psychological distress play central mediating roles in the association between chronotype and subjective perceptions of memory functioning, as shown in Table 4 and Figure 1.

## 4. Discussion

The present study aimed to clarify the interrelations between chronotype, sleep quality, psychological distress, and subjective memory complaints in a sample of Portuguese adults. By applying path analysis, this work advances existing research by testing a theoretically grounded model capable of capturing both direct and indirect associations among these constructs. Overall, the proposed model demonstrated a good and theoretically coherent fit to the data, supporting a circadian–affective framework linking biological timing preferences to subjective memory complaints.

The most salient finding was the identification of a sequential mediation pathway, whereby chronotype was indirectly associated with subjective memory complaints through sleep quality and psychological distress. Specifically, a more morning-oriented chronotype was associated with better perceived sleep quality, which in turn was linked to lower levels of distress and, sequentially, to fewer subjective memory complaints. This multistep pathway suggests that chronobiological predispositions influence subjective cognitive perception not directly, but through an interconnected cascade of sleep-related and emotional processes. Although eveningness has been independently associated with poorer sleep quality and greater distress (e.g., Antypa et al., 2016; Bauducco et al., 2020; Chauhan et al., 2024; Lan et al., 2025; Morris & Kountouriotis, 2024), and these factors have been linked to increased subjective memory complaints, the present study integrates these variables within a unified mediation model.

The specification of the sequential pathway (chronotype → sleep quality → psychological distress → subjective memory complaints) reflects a theoretically grounded psychobiological process. This sequential ordering was not arbitrary but reflects the temporal and regulatory hierarchy suggested in prior chronobiological and affective research (e.g., Fairchild & McDaniel, 2017; Kim et al., 2023; Morris & Kountouriotis, 2024). Although alternative directional models are theoretically plausible, the proposed ordering reflects the regulatory hierarchy described in chronobiological frameworks, where circadian predispositions are expected to influence sleep processes before shaping emotional and metacognitive appraisal. Circadian misalignment associated with eveningness may precede sleep disruption, particularly under socially imposed schedules, contributing to increased physiological distress. Sleep quality operates as a central regulatory mechanism within this system, given its established role in emotional modulation and cognitive functioning. Emotional distress, in turn, may influence metacognitive appraisal processes, biasing individuals toward more negative evaluations of their everyday cognitive performance. Emotional distress may also alter self-monitoring thresholds and heighten attentional sensitivity to minor lapses, thereby amplifying subjective memory perceptions independently of objective performance (e.g., Csábi et al., 2025; Dzierzewski, 2022). Within this framework, subjective memory complaints may represent affectively modulated perceptions of cognitive performance, influenced by sleep-related emotional processes rather than direct indicators of objective dysfunction. Consistent with broader psychobiological models, chronotype-related influences on cognition appear to operate through intermediary mechanisms rather than exerting isolated direct effects (Fairchild & McDaniel, 2017; Kim et al., 2023; Rucker et al., 2011). The absence of significant direct effects of chronotype on subjective memory complaints reinforces this interpretation and supports the view that circadian preference contributes indirectly to subjective cognitive experience via sleep and affective pathways.

The small-to-moderate magnitude of the bivariate associations is consistent with the multifactorial nature of the constructs under study. Rather than reflecting weak relationships, these effect sizes suggest that chronotype, sleep quality, and psychological distress represent partial—yet meaningful—contributors within a broader explanatory network influencing subjective memory complaints. This pattern aligns with the indirect structure observed in the path model, where effects operate through intermediary mechanisms rather than through strong direct associations.

The model accounted for a meaningful—though moderate—proportion of variance in subjective memory complaints. Given the inherently multifactorial nature of subjective cognitive perception, this level of explanatory power is theoretically expected. Subjective memory complaints have been shown to be influenced by multiple domains, including personality traits (e.g., neuroticism), cognitive reserve, physical health status, and lifestyle behaviors (e.g., Csábi et al., 2025). Thus, the present findings delineate one circadian–affective pathway without exhausting the broader explanatory architecture of subjective cognitive experience. Future research should integrate these additional domains within more comprehensive models.

The prominent role of psychological distress in the model is particularly noteworthy. Distress emerged as a predictor of subjective memory complaints, suggesting that individuals’ perceptions of memory difficulties may be more closely related to their emotional state and sleep quality (e.g., Basagni & Navarrete, 2025; Dzierzewski, 2022) than to chronotype per se. Anxiety, depression, and stress are known to influence cognitive self-evaluation processes and may amplify attentional focus on everyday lapses, even in the absence of objective impairment. Accordingly, subjective memory complaints should be interpreted within a broader affective and contextual framework rather than as unequivocal markers of cognitive decline (e.g., Csábi et al., 2025).

From a clinical and applied perspective, these findings carry important implications. Because subjective memory complaints often prompt initial clinical consultation (e.g., Dzierzewski, 2022), identifying modifiable intermediary mechanisms is of particular relevance. Interventions targeting sleep quality and circadian alignment—such as sleep hygiene education, chronotherapeutic approaches, and light-based strategies—may have downstream benefits for emotional well-being and perceived cognitive functioning. Complementary approaches focused on stress regulation and emotional resilience may further mitigate subjective memory complaints by addressing affective biases in cognitive appraisal. These results support integrative intervention strategies simultaneously addressing sleep and emotional processes.

Despite its contributions, several limitations must be acknowledged. First, the sample was predominantly female, young, and highly educated, which may limit generalizability. Women tend to report higher levels of psychological distress and subjective cognitive complaints, and the relatively young age of the sample suggests that subjective memory complaints are unlikely to reflect objective cognitive decline, reinforcing the relevance of affective and sleep-related mechanisms (e.g., Csábi et al., 2025; Cox et al., 2024). Additionally, high educational attainment may be associated with greater metacognitive awareness of everyday lapses. Future research should replicate these findings in more heterogeneous populations, including clinical samples and older adults.

Second, although structural equation modeling allows for theory-driven specification of directional pathways, the cross-sectional design precludes conclusions regarding temporal precedence and causality. Alternative models remain theoretically plausible, and the proposed pathway should therefore be interpreted as a theoretically informed explanatory model rather than evidence of causal sequencing. Longitudinal and experimental designs are necessary to clarify temporal dynamics.

Third, the exclusive reliance on self-report measures may introduce perception and recall biases. Because all variables were assessed using self-report instruments within the same assessment context, shared method variance may have inflated the observed associations, including the strength of the indirect effects identified in the mediation model. Therefore, the magnitude of the mediation pathway should be interpreted with caution.

Although internal consistency was satisfactory, future research would benefit from incorporating objective sleep measures (e.g., actigraphy), neuropsychological assessment of memory, and biological markers of circadian function. Multimethod approaches would allow clearer differentiation between subjective perception and objective cognitive functioning.

## 5. Conclusions

This study highlights the importance of chronotype as a distal factor linked to psychological and cognitive well-being primarily through its associations with sleep quality and psychological distress. The findings indicate that eveningness is associated with poorer sleep quality, which in turn predicts higher levels of distress, thereby shaping individuals’ subjective perceptions of memory functioning. The proposed sequential mediation model offers a theoretically grounded and nuanced psychobiological explanation of how circadian preferences translate into subjective cognitive experiences.

By addressing an underexplored aspect of circadian research—subjective memory complaints—this study contributes original and relevant insights to the literature and underscores the relevance of sleep and emotional processes as key intervention targets. Despite its limitations, the present investigation provides a conceptually robust framework for future research and supports the development of personalized intervention strategies aimed at improving sleep quality and reducing distress in order to promote cognitive and emotional well-being among adult populations.

## Figures and Tables

**Table 1:** Sociodemographic and Chronotype Characteristics of the Sample (*N* = 382).

*Variable*	*n*	%
**Sex**		
Female	341	89.3
Male	41	10.7
**Educational level**		
Post-secondary non-tertiary education	226	59.2
Bachelor’s degree	12	3.1
Master’s degree	116	30.4
Doctoral degree	20	5.2
Lower than post-secondary education	8	2.1
**Chronotype (MEQ classification)**		
Morning-type	78	20.4
Intermediate-type	180	47.1
Evening-type	124	32.5

***Note:*** Chronotype (or morningness–eveningness) classification was based on established MEQ cut-off scores (da Silva et al., 2002).

**Table 2:** Descriptive statistics for the main study variables.

*Variable*	Skewness	Kurtosis	Mean	Standard Deviation	Min–Max
Morningness–eveningness	−0.31	0.07	46.13	8.65	19–67
Anxiety	1.45	1.85	4.07	4.32	0–20
Stress	0.59	−0.21	7.10	4.66	0–21
Depression	1.36	1.39	4.35	4.44	0–21
Subjective Memory Complaints	0.62	−0.17	17.20	9.87	0–45
Sleep Quality	0.84	0.35	6.14	3.21	0–17

**Table 3:** Pearson Correlation Matrix among the Variables.

*Variable*	Morningness–Eveningness	Anxiety	Stress	Depression	Subjective Memory Complaints
Anxiety	−0.071				
Stress	−0.103 *	0.770 ***			
Depression	−0.174 **	0.677 ***	0.704 ***		
Subjective Memory Complaints	−0.099	0.363 ***	0.412 ***	0.405 ***	
Sleep Quality	−0.229 ***	0.325 ***	0.370 ***	0.437 ***	0.255 ***

***Note***: * *p* < 0.05. ** *p* < 0.01. *** *p* < 0.001.

**Table 4:** Direct and Indirect Effects that emerged in the Path Analysis.

Path	*β*	*Standard Error*	*p*	*IC 95% (BC)*
**Direct Effects**				
Chronotype → Sleep Quality	−0.229	0.052	<0.001	[−0.326; −0.125]
Chronotype → Distress	−0.030	0.048	0.519	[−0.127; 0.062]
Sleep Quality → Distress	0.426	0.052	<0.001	[0.321; 0.525]
Sleep Quality → Subjective Memory Complaints	0.062	0.054	0.224	[−0.043; 0.168]
Chronotype → Subjective Memory Complaints	−0.030	0.045	0.499	[−0.120; 0.058]
**Indirect Effects**				
Chronotype → Sleep Quality → Distress	−0.097	0.026	0.000	[−0.153; −0.052]
Chronotype → Sleep Quality → Subjective Memory Complaints	−0.069	0.027	0.006	[−0.126; −0.017]
Chronotype → Sleep Quality → Distress → Subjective Memory Complaints	−0.048	0.015	<0.001	[−0.084; −0.024]

***Note***: The coefficients presented are standardized.

## Data Availability

The data presented in this study are available on request from the corresponding author (PFSR). The data are not publicly available due to privacy or ethical restrictions.

## Notes

1 Although chronotype and morningness–eveningness are related constructs associated with biological rhythms and diurnal time preferences, chronotype represents a broader concept encompassing underlying physiological processes, whereas morningness–eveningness refers more specifically to behavioral tendencies toward morning or evening activities. In the present manuscript, these constructs are treated as synonymous.

2 Although the MEQ allows participants to be classified into morning, evening, and intermediate chronotypes, the present study used the total questionnaire score as a continuous measure of morningness–eveningness, with higher scores indicating a tendency for morningness.
